# A phase 4, open-label, multicenter study of the safety and efficacy of agalsidase beta in Chinese patients with Fabry disease

**DOI:** 10.1186/s13023-025-03950-7

**Published:** 2025-08-04

**Authors:** Hong Ren, Wei Zhang, Yan Ouyang, Junhong Guo, Hong Xu, Jie Ma, Xiaoping Luo, Xiaoxia Pan, Yun Yuan, Wei Zhang, Qian Shen, Bin Li, Qiqi Feng, Shi Liu, Nan Chen

**Affiliations:** 1 Department of Nephrology, Institute of Nephrology, Ruijin Hospital, The Medical School of Shanghai Jiao Tong University, No. 197 Ruijin Er Road, Shanghai, China; 2 Department of Neurology, Peking University First Hospital, No. 8 Xishiku Street, Beijing, China; 3 Department of Neurology, First Hospital of Shanxi Medical University, No. 85 South Jiefang Road, Taiyuan, Shanxi China; 4 Department of Nephrology, Children’s Hospital of Fudan University, No. 399 Wanyuan Road, Shanghai, China; 5 Department of Nephrology, Peking Union Medical College Hospital, No.1 Shuaifuyuan Wangfujing Dongcheng District, Beijing, China; 6 Department of Pediatrics, Tongji Hospital, Tongji Medical College, Huazhong University of Science and Technology, No.1095 Jiefang Avenue, Wuhan, Hubei China; 7 Medical Department of Rare Disease & Rare Blood Disorders, Sanofi, No. 1228 Middle Yan’an Road, Shanghai, China

**Keywords:** Agalsidase beta, COVID-19, Fabry disease, Infusion-associated reactions, Kidney function

## Abstract

**Background:**

This is the first phase 4 study evaluating safety and efficacy of enzyme replacement therapy (ERT) in Chinese patients with Fabry disease, and exploring the impact of COVID-19 infection on the prognosis of Fabry disease under ERT.

**Methods and results:**

Eligible patients received an infusion of agalsidase beta (1.0 mg/kg/2w) for up to 48 weeks. The primary endpoint was the safety of agalsidase beta. The endpoints of efficacy included changes in plasma globotriaosylceramide (GL-3), globotriaosylsphingosine (Lyso-GL-3), symptoms and estimated glomerular filtration rate (eGFR) from baseline to week 48. A post-hoc subgroup analysis was conducted by age group (< 30 years and ≥ 30 years) and in patients with or without COVID-19 infection. All 22 patients completed the study and 14 of them were infected by COVID-19. Treatment-related adverse events (AEs) and infusion-associated reactions (IARs) were reported in 8 participants (36.4%). Mean plasma GL-3 (-34.6%) and Lyso-GL-3 (-60.3%) levels decreased from baseline to week 48. Thirteen participants (59.1%) experienced improved specific symptoms at week 48. There were no meaningful changes in eGFR during the study, and the overall population showed an annual eGFR slope of 0.43 mL/min/1.73 m^2^/year (95% CI: -5.95 to 6.82). In the subgroup analysis, the reductions in plasma GL-3 and Lyso-GL-3 levels, improvement in symptoms, and attenuation of eGFR decline after 48 weeks of treatment were generally greater in patients aged < 30 years (n = 11) than in patients aged ≥ 30 years (n = 11), and less pronounced in the COVID-19 infected group (n = 14) than in the uninfected group (n = 8).

**Conclusions:**

This study demonstrates that agalsidase beta is safe and effective in Chinese patients with Fabry disease, and suggestes that COVID-19 infection may potentially impact the renal prognosis for Fabry disease.

Trial registration: ClinicalTrials.gov, NCT05054387. Registered 09 September 2021, https://clinicaltrials.gov/study/NCT05054387

**Supplementary Information:**

The online version contains supplementary material available at 10.1186/s13023-025-03950-7.

## Introduction

Fabry disease, a rare X-linked lysosomal storage disorder, arises from mutations in the alpha galactosidase (*GLA*) gene, responsible for encoding the lysosomal enzyme alpha-galactosidase A (α-Gal A, enzyme commission no. 3.2.1.22 [1]). Pathogenic variations in the *GLA* gene lead to reduced or altered enzymatic activity, causing the progressive accumulation of globotriaosylceramide (GL-3) and its deacylated form, globotriaosylsphingosine (Lyso-GL-3), in affected cells across various tissues [2, 3]. This accumulation triggers a series of clinical manifestations, such as neuropathic pain, angiokeratomas, sweating abnormalities, gastrointestinal manifestations, eye lesions and hearing abnormalities. As the disease progresses, multiorgan damage gradually aggravates, which can eventually lead to life-threatening complications in the kidneys, heart, and cerebrovascular system, as well as premature death [4]. Fabry disease exhibits classic and late-onset types based on clinical manifestations. In classic Fabry disease which mainly affects males, the enzyme activity is absent or significantly reduced. Chronic neuropathic pain, impaired sweat function, and angiokeratomas typically emerge in childhood, with potential kidney, heart, and central nervous system involvement in adulthood. Classic patients generally experience more severe clinical manifestations, a worse prognosis, and a higher risk of clinical events. Late-onset Fabry disease phenotype presents with partially preserved enzymatic activity showcasing cardiac symptoms and kidney involvement in the fourth to seventh decades of life, reflecting delayed onset and slower disease progression [5–7]. The onset and severity of symptoms in women primarily depends on the random inactivation pattern of one of the two X chromosomes. [8]

Enzyme replacement therapy (ERT) with exogenous human α-Gal A has been the primary treatment for Fabry disease since 2001, recommended by various guidelines for the management of patients with Fabry disease [7, 9, 10]. Three ERT preparations are globally approved for marketing: agalsidase beta, agalsidase alfa and pegunigalsidase alfa. However, agalsidase beta is the only ERT approved by the United States Food and Drug Administration (FDA) for Fabry disease patients aged 2 years and older, while pegunigalsidase alfa is approved by FDA for the treatment of adult patients. Numerous studies confirm that agalsidase beta promotes the decomposition of GL-3, reduces the accumulation of GL-3 and Lyso-GL-3 in organ tissues, and is associated with a significantly lower incidence of renal, cardiovascular, and cerebrovascular events compared to untreated patients [11, 12].

Following approval of agalsidase beta (Fabrazyme®, Sanofi, 1 mg/kg every other week) for Fabry disease in the Chinese mainland in December 2019, a post-marketing surveillance (PMS) study was conducted to evaluate the safety and efficacy of agalsidase beta in Chinese population as required by the Chinese health authority. Before current study, there was a lack of data on the safety and efficacy of agalsidase beta in Chinese patients with Fabry disease. Moreover, this multicenter study on agalsidase beta marks the first phase 4 evaluation of safety and efficacy of ERT in Chinese patients with Fabry disease. The study was conducted between 2021 and 2023 during the COVID-19 pandemic. We also explored the impact of COVID-19 infection on the prognosis of Fabry disease under ERT.

## Materials and methods

### Study design

This study (NCT05054387) was conducted in accordance with the protocol and consensus ethical principles derived from international guidelines including the Declaration of Helsinki, and the International Council for Harmonisation (ICH) guidelines for Good Clinical Practice (GCP), in addition to all applicable laws, rules, and regulations. The protocol and its amendments were reviewed and approved by the Institutional Review Board / Independent Ethics Committee (IRB/IEC) before study initiation. The genetic data reported in this paper have been deposited in the OMIX, China National Center for Bioinformation / Beijing Institute of Genomics, Chinese Academy of Sciences (https://ngdc.cncb.ac.cn/omix: accession no. OMIX005209). [13, 14]

**Fig. 1:**
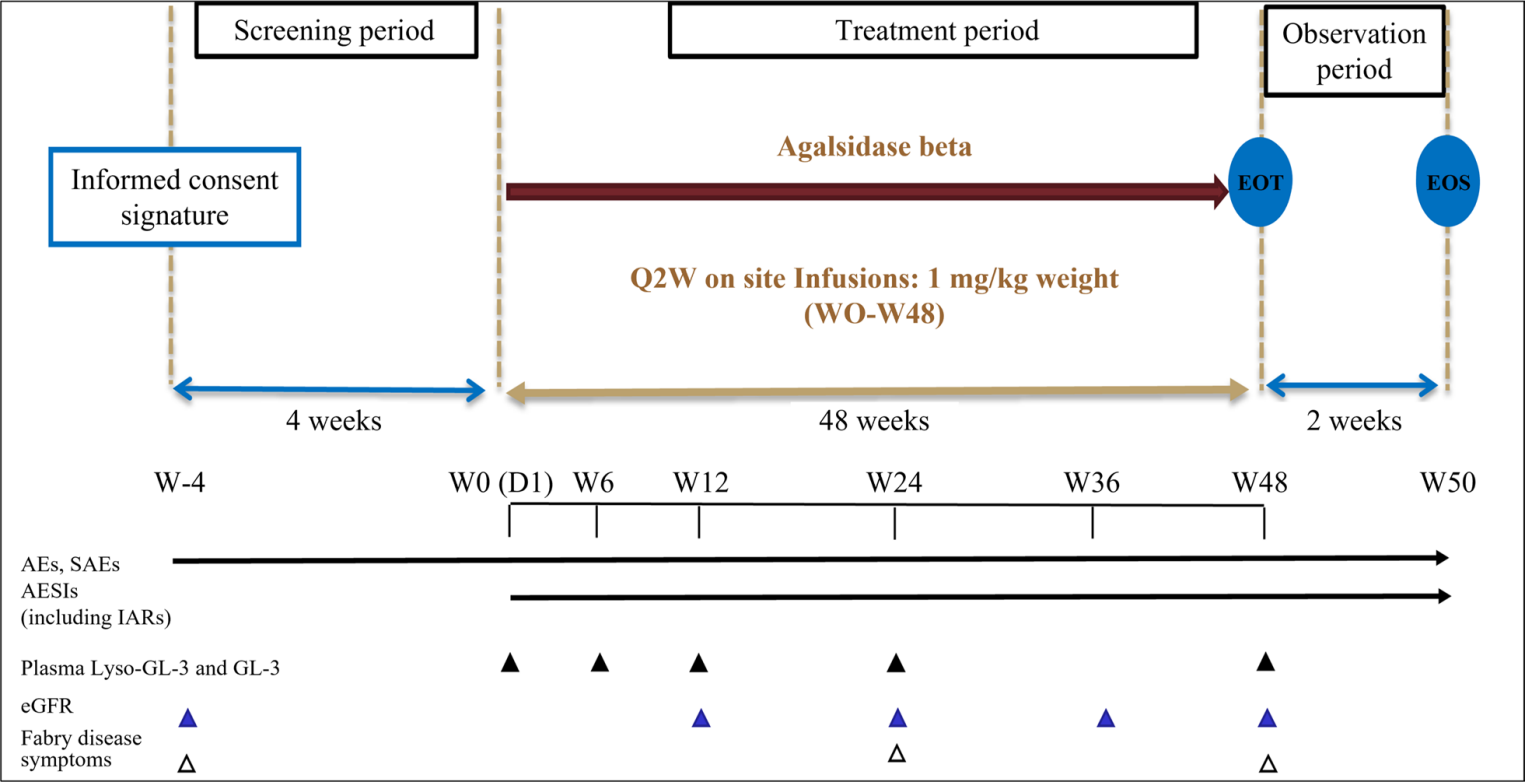
Graphical study design. *AE*, adverse event; *AESI*, adverse event of special interest; *D*, day; *eGFR*, estimated glomerular filtration rate; *EOT*, end-of-treatment; *EOS*, end-of-study; *GL-3*, globotriaosylceramide; *IAR*, infusion associated reaction; *Lyso-GL-3*, globotriaosylsphingosine; *Q2W*, every 2 weeks; *SAE*, serious adverse event; *W*, week

This was a 54-week, phase 4, open label, single arm clinical trial designed to evaluate the safety and efficacy of agalsidase beta as ERT in Chinese patients with Fabry disease. Each patient’s study period spanned 54 weeks, including 4 weeks of screening, 48 weeks of treatment, and 2 weeks of post-study treatment observation (Fig. 1). Eligible patients with Fabry disease received agalsidase beta via infusion at a dose of 1.0 mg/kg every 2 weeks for up to 48 weeks. The initial infusion rate was limited to no more than 0.25 mg/min (15 mg/hour) to minimize the potential occurrence of infusion-associated reactions (IARs). For patients weighing 30 kg or greater, after patient tolerance to the infusion was well established, the infusion rate was increased in increments of 0.05 to 0.08 mg/min (increments of 3 to 5 mg/hour) with each subsequent infusion. The minimum infusion duration was 1.5 h (based on individual patient tolerability). The maximum infusion rate for patients weighing less than 30 kg, was 0.25 mg/minute (15 mg/hour). The treatment compliance was defined as the number of infusions the participant actually received divided by the total number of infusions planned throughout the trial.

### Patients

All eligible participants in this study were Chinese individuals aged 8 years and older with a confirmed diagnosis of Fabry disease based on documented plasma or leukocyte α-Gal A activity below the laboratory’s reference range, and/or documented diagnosis by genotyping. None of the patients had received prior treatment with agalsidase beta or agalsidase alfa. Inclusion criteria stipulated that patients must exhibit one or more symptoms and signs consistent with manifestations of Fabry disease. Key exclusion criteria were patients who had undergone kidney transplantation, clinically significant organ disease (except for symptoms related to Fabry disease) at the discretion of investigators, and current evidence of kidney failure or insufficiency defined by an estimated glomerular filtration rate (eGFR) < 30 mL/min/1.73 m^2^. Patients were permitted to receive concomitant medications during the study intervention period, except for α-Gal A inhibitors such as chloroquine, amiodarone, benzoquinone, or gentamicin.

A total of 24 patients provided informed consent and underwent screening, of which 22 patients were enrolled at 6 study sites in China. Two patients were excluded due to screen failure. The study period was from the first patient’s first visit on October 15, 2021, to the last patient’s last visit on March 9, 2023. The entire study was conducted during the COVID-19 pandemic, which to some extent might have impacted the study procedures.

Enrolled patients were classified by the phenotypes of Fabry disease including classic and late-onset types.

### Safety assessments

The primary objective of this study was to evaluate the safety and tolerability of agalsidase beta (1 mg/kg, IV, Q2W) in Chinese patients with Fabry disease. The primary endpoints of safety were the incidence of treatment-emergent adverse events (TEAEs), treatment-emergent serious adverse events (SAEs), and adverse events of special interest (AESI) including IARs; the change in clinical laboratory parameters (hematology [red blood cells, platelets, coagulation, and white blood cells] and biochemistry [metabolism, kidney function, and liver function]), vital signs (blood pressure, heart rate, weight, temperature and respiratory rate), and Electrocardiogram (ECG) parameters (heart rate, PR interval, QT interval, QTc correction method unspecified, QRS duration, and RR interval) throughout the treatment emergent period. TEAEs are defined as adverse events that develop, worsen, or become serious during the treatment-emergent period. IAR is defined as AE that occurs during the infusion or within 24 h after the start of infusion and is considered as related or possibly related to the study intervention by the Investigator or Sponsor excluding laboratory, ECG, and echocardiography (ECHO) abnormalities. An event occurring ≥ 24 h after the start of an infusion may be deemed an IAR if it could be considered as a delayed reaction by the Investigator or Sponsor. Patients experiencing an IAR who remained eligible for the study were allowed to subsequently reduce infusion rate with pretreatment medications such as non-steroidal anti-inflammatory medicinal products, antihistamines and/or corticosteroids per Investigator’s judgement and guidance from Chinese package insert. Pretreatment medications were administered at least 1 h prior to infusion in order to obtain a maximum effect at the time of infusion. Although anti-drug antibodies (ADAs) could have an impact on the safety profile of enzyme replacement therapy, unfortunately, the assessment of ADAs was not incorporated into the study protocol.

### Assessments of clinical efficacy

Clinical efficacy was assessed through multiple endpoints, including: 1) Absolute change and percent change in plasma GL-3 (normal range: 1.1–3.2 ug/mL) and Lyso-GL-3 (normal range: 0.22–0.489 ng/mL) using a validated liquid chromatography-tandem mass spectrometry from baseline to week 6, week 12, week 24, and week 48 (see Additional file 1); 2) Changes in Fabry disease symptoms (including angiokeratoma, sweating, chronic abdominal pain, level of activity, exercise tolerance and heat tolerance, headache, and tinnitus) from baseline to week 24 and week 48; 3) Absolute change and slope of eGFR calculated using the Chronic Kidney Disease Epidemiology Collaboration (CKD-EPI) equation for adults (≥ 18 years) [15] and the Bedside Schwartz equation for children ((8 ≤ age < 18 years) [16] from baseline to week 12, week 24, week 36, and week 48.

In addition, a post-hoc subgroup analysis was conducted by age group (< 30 years and ≥ 30 years) and in patients with or without COVID-19 infection. The differences in plasma GL-3, Lyso-GL-3, eGFR and symptom improvement between these subgroups were analyzed.

Cardiac outcome evaluations were not considered in this study, as the short follow-up duration and the low number of patients with cardiac abnormalities were insufficient to achieve the statistical power required for such assessments.

### Statistical analysis

Descriptive statisics were applied in this study to summarize the safety and efficacy parameters. The annualized eGFR slope was estimated using a linear mixed effect model with random intercept and slope, accouting for the individual eGFR changes over time. The spaghetti plots were used to display the changes in plasma GL-3 and Lyso-GL-3 levels, as well as eGFR values over time of each subject. All analyses were conducted in the exposed population, defined as individuals who provided informed consent and received at least one dose of agalsidase beta. The baseline value was defined as the last available value before the first dose of agalsidase beta. No formal statistical hypothesis testing was planned in this study. The sample size was determined by the regulatory requirement and feasibility considerations.

## Results

### Characteristics of the patients

All 22 patients enrolled in the study received the intervention and completed the treatment. The entire study was conducted during the COVID-19 pandemic, during which 14 patients were infected. The mean duration of study intervention exposure was 48.6 ± 8.4 weeks, and 20 patients (90.9%) received study intervention exposure for over 48 weeks. The mean percentage of study treatment compliance with agalsidase beta administration was 74.9 ± 19.2%, with a total of 11 patients (50%) achieving an overall compliance of ≥ 80%. All participants experienced at least one critical or major protocol deviation, primarily due to the impact of the COVID-19 pandemic. The most frequently reported deviations were related to assessments/procedures (100%) and investigational medicinal product management (86.4%), but these did not affect the overall safety outcomes.

At the initiation of ERT, the mean age of the 22 participants was 29.0 ± 13.5 years old (range: 10 to 60), with 18 patients (81.8%) aged 18 to 60 years (Tables 1 and 2). Eighteen patients (81.8%) were male and 4 (18.2%) were female. At baseline, 20 patients (90.9%) had decreased level of α-Gal A activity, while 2 female patients (9.1%) had normal level of α-Gal A activity. The mean plasma levels of GL-3 and Lyso-GL-3 were 6.91 ± 2.81 ug/mL and 66.27 ± 38.99 ng/mL at baseline, respectively. The mean plasma GL-3 and Lyso-GL-3 levels in male patients were well above the normal range, while mean plasma GL-3 levels in female patients were within the normal range and mean Lyso-GL-3 levels were lower than that in male patients, despite being above the upper limit of the normal range. Various *GLA* gene mutations were detected, with the majority being missense mutations (15/22, 68.2%). Twenty patients (90.9%) were categorized as having the classic phenotype of Fabry disease (17 males and 3 females), while 2 patients (9.1%) were categorized as having the late-onset phenotype (one for each male and female). The mean eGFR for all patients was 115.6 ± 37.3 mL/min/1.73m^2^ at baseline, and eGFR levels were almost similar between male and female patients. Overall, 17 patients (77.3%) reported at least one medical or surgical history, and 17 patients (77.3%) had received prior medications before their first study intervention (Table 1).

**Table 1:** Patient demographics and baseline characteristics

Variable	Total	Male	Female
N (%)	22 (100)	18 (81.8)	4 (18.2)
Age (years), mean ± SD	29.0 ± 13.5	26.7 ± 11.0	39.3 ± 20.5
Weight (kg), mean ± SD	58.4 ± 15.1	59.6 ± 15.8	53.1 ± 11.7
Height (cm), mean ± SD	167.9 ± 11.6	169.2 ± 12.2	162.0 ± 6.7
Phenotype, n(%)			
Classic	20 (90.9)	17 (94.4)	3 (75.0)
Late-onset	2 (9.1)	1 (5.6)	1 (25.0)
Mutations, n(%)			
Missense	15 (68.2)	12 (66.7)	3 (75.0)
Nonsense	5 (22.7)	5 (27.8)	0 (0)
Frameshift	1 (4.5)	0 (0)	1 (25.0)
Deletion	1 (4.5)	1 (5.6)	0 (0)
Patients with abnormal α-Gal A activity^a^, n(%)	20 (90.9)	18 (100.0)	2 (50.0)
GL-3 (ug/mL), mean ± SD	6.91 ± 2.81	7.77 ± 2.32	3.07 ± 0.77
Patients with abnormal level of GL-3^b^, n(%)	18 (81.8)	17 (94.4)	1 (25.0)
Lyso-GL-3 (ng/mL), mean ± SD	66.27 ± 38.99	79.44 ± 29.39	7.01 ± 4.68
Patients with abnormal level of Lyso-GL-3^c^, n(%)	22 (100.0)	18 (100.0)	4 (100.0)
eGFR (mL/min/1.73m^2^), mean ± SD	115.6 ± 37.3	116.2 ± 37.8	112.7 ± 40.8
Medical history, n(%)	17 (77.3)	14 (77.8)	3 (75.0)
Hypertension	5 (22.7)	3 (16.7)	2 (50.0)
Chronic kidney disease	4 (18.2)	3 (16.7)	1 (25.0)
Hyperhomocysteinaemia	3 (13.6)	2 (11.1)	1 (25.0)
Thyroid mass	3 (13.6)	1 (5.6)	2 (50.0)
Glomerulonephritis chronic	2 (9.1)	2 (11.1)	0 (0)
Appendicitis	2 (9.1)	1 (5.6)	1 (25.0)
Hypothyroidism	2 (9.1)	1 (5.6)	1 (25.0)
Folate deficiency	2 (9.1)	2 (11.1)	0 (0)
Cardiac hypertrophy	2 (9.1)	1 (5.6)	1 (25.0)
Proteinuria	2 (9.1)	2 (11.1)	0 (0)
Any prior medications, n(%)	17 (77.3)	14 (77.8)	3 (75.0)
Analgesics	9 (40.9)	7 (38.9)	2 (50.0)
Agents acting on the renin-angiotensin system	8 (36.4)	7 (38.9)	1 (25.0)
Antiepileptics	7 (31.8)	7 (38.9)	0 (0)
Unspecified herbal and traditional medicine	6 (27.3)	6 (33.3)	0 (0)
Psycholeptics	5 (22.7)	5 (27.8)	0 (0)
Urologicals	5 (22.7)	5 (27.8)	0 (0)
Beta blocking agents	2 (9.1)	2 (11.1)	0 (0)
Cardiac therapy	2 (9.1)	1 (5.6)	1 (25.0)
Antithrombotic agents	2 (9.1)	1 (5.6)	1 (25.0)

*α-Gal A*, Alpha-galactosidase A; *eGFR*, estimated glomerular filtration rate; *GL-3*, globotriaosylceramide; *Lyso-GL-3*, globotriaosylsphingosine; *N*, number; *SD*, Standard deviation

^a^The α-Gal A activity data for each patient were derived from previous diagnostic history, and the patients were not retested for α-Gal A activity in this study

^b^Normal Reference Range: 1.1–3.2 ug/mL

^c^Normal Reference Range: 0.22–0.489 ng/mL

**Table 2:** Baseline characteristics of 22 Chinese patients with Fabry disease

Patient	Age (years)	Sex	Phenotype	*GLA* Gene mutations
01	39	M	Late-onset	c.613C > T, p.Pro205Ser
02	48	M	Classic	c.776C > T, p.Pro259Leu
03	20	M	Classic	c.334C > T, p.Arg112Cys
04	32	M	Classic	c.1115T > C, p.Leu372Pro
05	60	F	Late-onset	c.194G > A, p.Ser65Asn
06	21	M	Classic	c.658C > T, p.Arg220*
07	27	M	Classic	c.678G > A, p.Trp226*
08	15	M	Classic	c.334C > T, p.Arg112Cys
09	33	M	Classic	c.132G > T, p.Trp44Cys
10	48	F	Classic	c.266T > G, p.Leu89Arg
11	43	M	Classic	c.901C > T, p.Arg301*
12	20	M	Classic	c.463G > C, p.Asp155His
13	31	M	Classic	c.803_806del, p.Leu268*
14	10	M	Classic	c.100A > C, p.Asn34His
15	10	M	Classic	c.100A > C, p.Asn34His
16	12	F	Classic	c.100A > C, p.Asn34His
17	19	M	Classic	c.902G > A, p.Arg301Gln
18	21	M	Classic	Exon 2del
19	35	M	Classic	c.1021G > A, p.Glu341Lys
20	37	F	Classic	c.1033_1034delTC, p.Ser345fs
21	36	M	Classic	c.101A > G, p.Asn34Ser
22	21	M	Classic	c.1196G > A, p.Trp399*

*F*, female; *M*, male

### Safety

All patients experienced at least one AE, and all identified events in this study were TEAEs. The most frequently reported AEs were related to COVID-19 (14/22, 63.6%). The majority of AEs in the study were of mild to moderate in severity. SAEs were reported in two patients (9.1%), and these events were not related to the study drug. The SAE of tension headache was reported in one patient (4.5%) with an outcome of recovered/resolved, while another patient (4.5%) reported end stage renal disease (ESRD) as an SAE with an outcome of recovering/resolving. No AEs led to permanent treatment discontinuation or death during the study (Table 3).

**Table 3:** Overview of adverse event profile

n (%)	Agalsidase beta (N = 22)
Patients with any AEs	22 (100)
Most frequently reported (≥ 10%)	
COVID-19	14 (63.6)
Influenza	4 (18.2)
Upper respiratory tract infection	4 (18.2)
Dizziness	3 (13.6)
Nausea	3 (13.6)
Pruritus	3 (13.6)
Feeling hot	3 (13.6)
Pyrexia	3 (13.6)
Patients with any SAE	2 (9.1)
Tension headache	1 (4.5)
End stage renal disease	1 (4.5)
Patients with any AEs leading to death	0
Patients with any AEs leading to permanent treatment discontinuation	0
Patients with any treatment-related AEs	8 (36.4)
Most frequently reported (≥ 5%)	
Feeling hot	3 (13.6)
Pyrexia	3 (13.6)
Nausea	2 (9.1)
Pruritus	2 (9.1)
Pain in extremity	2 (9.1)
Chest discomfort	2 (9.1)
Patients with any AESI	8 (36.4)
Patients with any IARs	8 (36.4)

*AE*, adverse event; *SAE*, serious adverse events; *AESI*, adverse events of special interest; *IAR*, infusion associated reaction; *N*, number

Eight patients (36.4%) reported treatment-related AEs. AESIs were reported in 8 patients (36.4%), and all these AESIs were IARs (Supplemental Table S1 [see Additional file 1]). IARs were reported in 7 (including 6 classic and 1 late-onset) out of 18 male patients and 1 (late-onset) out of 4 female patients. The first occurrence of IARs were predominantly within 13 infusions, and the incidence of IARs decreased gradually over time. None of these IARs were considered serious or resulted in permanent treatment discontinuation.

Besides, there were no considerable differences in the mean changes of clinical laboratory values and vital signs at week 48 compared to baseline.

### Efficacy

#### Plasma GL-3 and Lyso-GL-3

**Fig. 2:**
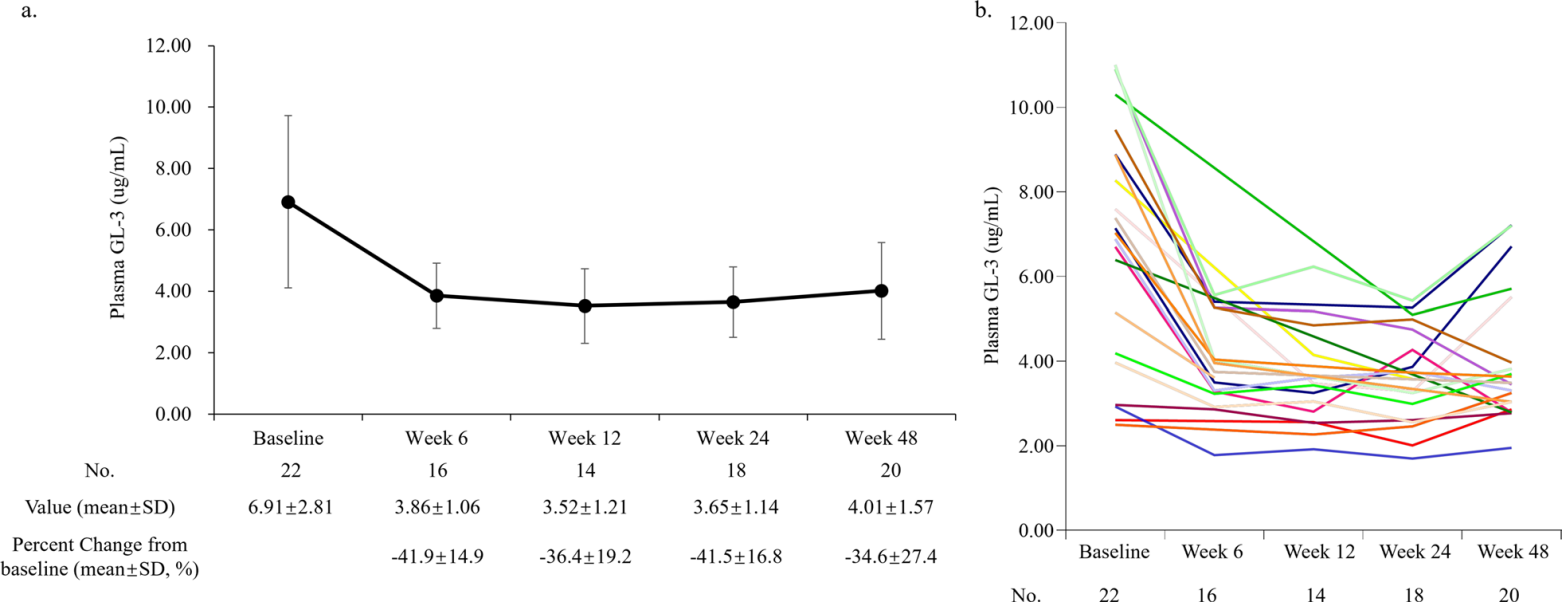
**a** The mean value for plasma GL-3 from baseline to week 48; **b** Spaghetti plot of plasma GL-3 changes from baseline to week 48 in 22 patients. *GL-3*, globotriaosylceramide; *No.*, number; *SD*, standard deviation

**Fig. 3:**
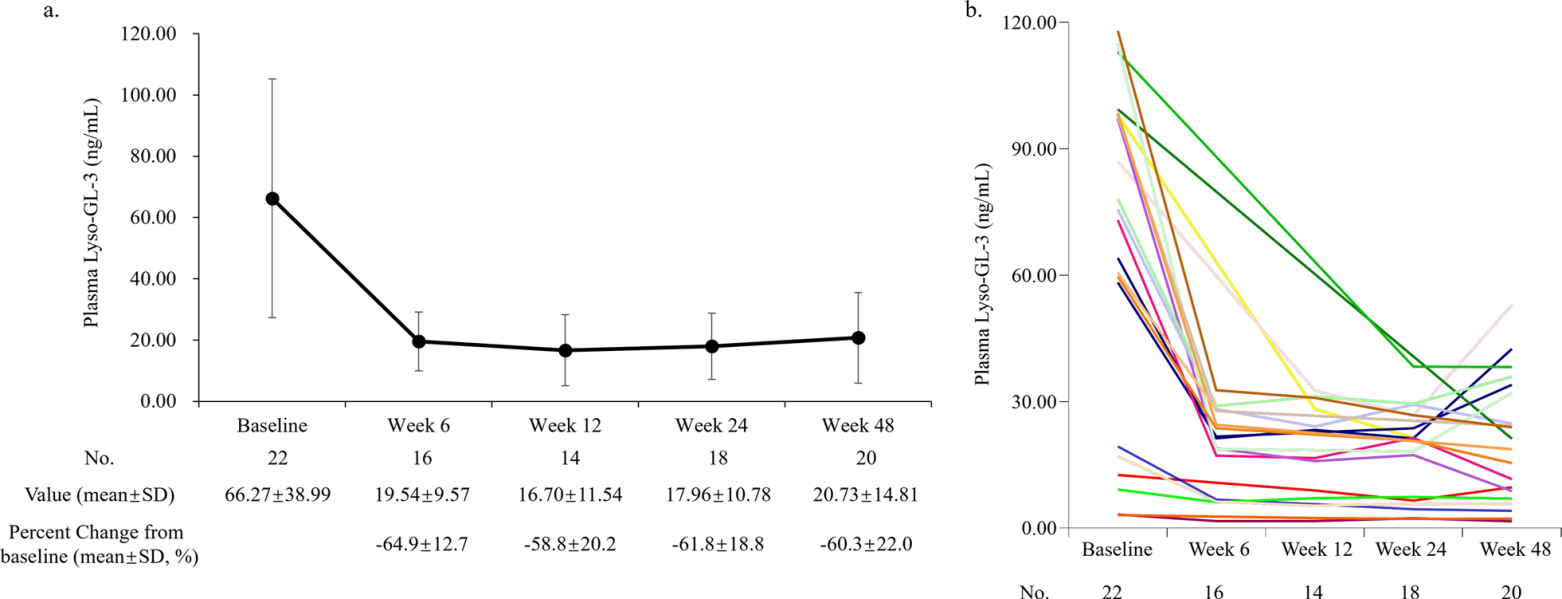
**a** The mean value for plasma Lyso-GL-3 from baseline to week 48; **b** Spaghetti plot of plasma Lyso-GL-3 changes from baseline to week 48 in 22 patients. *Lyso-GL-3*, globotriaosylsphingosine; *No.*, number; *SD*, standard deviation

The study intervention led to a notable reduction in plasma GL-3 and Lyso-GL-3 values during the treatment period compared to baseline. At week 48, the mean plasma GL-3 value was 4.01 ± 1.57 ug/mL, showing a decrease from the baseline value of 6.91 ± 2.81 ug/mL (Fig. 2a and b, and Supplemental Figure S1a and S1b [see Additional file 1]). The mean percentage reduction of plasma GL-3 from baseline ranged from -34.6 ± 27.4% to -41.9 ± 14.9% during week 6 through week 48. Similarly, the mean plasma Lyso-GL-3 value at week 48 was 20.73 ± 14.81 ng/mL, decreased from the baseline value of 66.27 ± 38.99 ng/mL (Fig. 3a and b, and Supplemental Figure S2a and S2b [see Additional file 1]). The mean percentage reduction of plasma Lyso-GL-3 from baseline ranged from -58.8 ± 20.2% to -64.9 ± 12.7% during week 6 through week 48.

In the post-hoc analysis, classic patients (n = 20) showed mean plasma GL-3 and Lyso-GL-3 levels of 7.28 ± 2.67 ug/mL and 71.89 ± 36.17 ng/mL at baseline, and 4.11 ± 1.63 ug/mL and 22.59 ± 14.43 ng/mL at week 48, respectively. For late-onset patients (n = 2), the mean plasma GL-3 and Lyso-GL-3 levels were 3.24 ± 1.04 ug/mL and 10.03 ± 9.86 ng/mL at baseline, and 3.15 ± 0.15 ug/mL and 4.01 ± 2.50 ng/mL at week 48, respectively. The reduction was more pronounced for classic male patients (GL-3: 7.99 ± 2.19 ug/mL at baseline and 4.31 ± 1.71 ug/mL at week 48, -46.0 ± 18.4%; Lyso-GL-3: 83.11 ± 25.69 ng/mL at baseline and 25.89 ± 13.43 ng/mL at week 48, -67.8 ± 17.6%).

#### Symptom assessment

After 24 weeks of treatment with agalsidase beta, 36.4% of patients (8/22) reported improved Fabry disease symptoms, while 63.6% (14/22) showed no change in symptoms. By week 48, the proportion of patients experiencing improvement increased to 59.1% (13/22), and the proportion of patients with unchanged symptoms decreased to 36.4% (8/22). None of the patients exhibited worsening of symptoms over the course of the study.

#### Kidney function (eGFR)

**Fig. 4:**
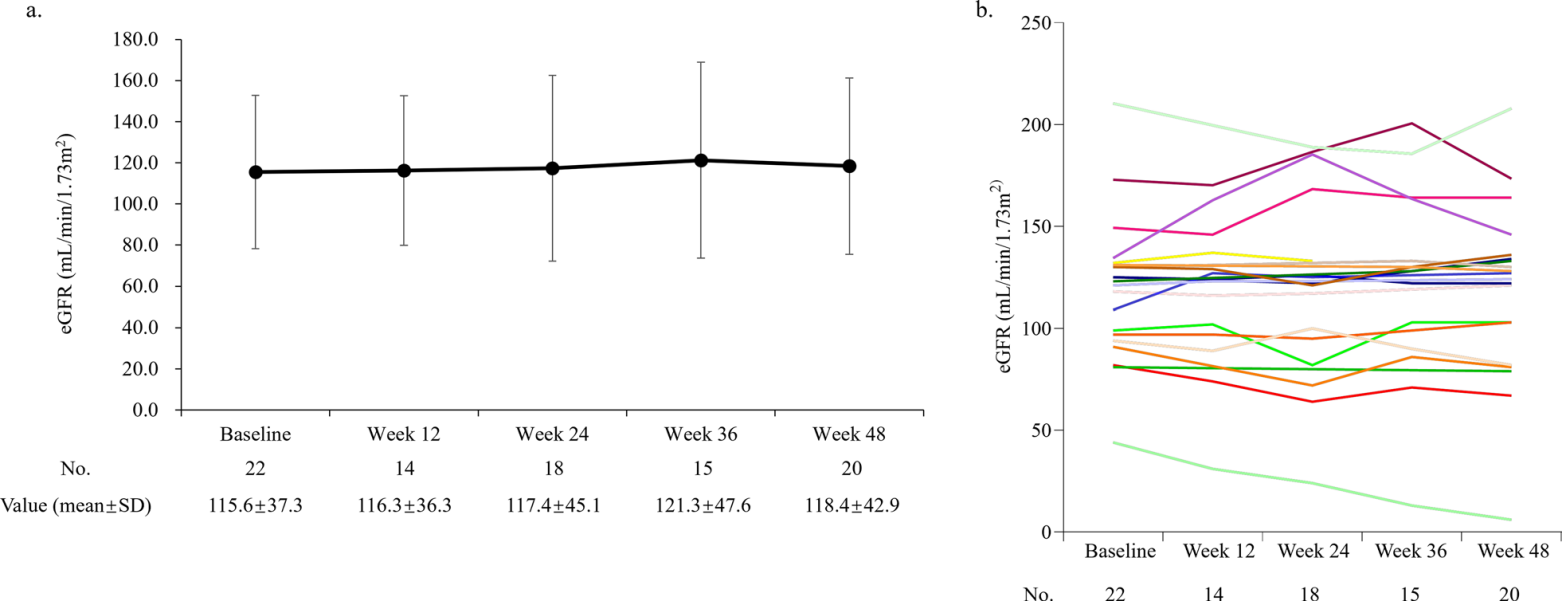
**a**. The mean value for eGFR from baseline to week 48; **b**. Spaghetti plot of eGFR changes from baseline to week 48 in 22 patients. *eGFR*, estimated glomerular filtration rate; *No.*, number; *SD*, standard deviation

There was no clinically meaningful change in the mean eGFR during the treatment period. The mean eGFR was 115.6 ± 37.3 mL/min/1.73m^2^ at baseline and 118.4 ± 42.9 mL/min/1.73m^2^ at week 48, reflecting a change of 0.0 ± 12.4 mL/min/1.73m^2^ from baseline to week 48 (Fig. 4a and b, and Supplemental Figure S3a, S3b and S4 [see Additional file 1]). The overall population had an annual eGFR slope of 0.43 mL/min/1.73 m^2^/year (95% CI: -5.95 to 6.82), with male patients having an annual eGFR slope of - 0.17 mL/min/1.73 m^2^/year (95% CI: -7.71 to 7.37).

#### Post-hoc subgroup analysis by age and COVID-19 infection status

In the post-hoc analysis based on age, participants were categorized into two groups: the younger group with age < 30 years (n = 11) and the older group with age ≥ 30 years (n = 11), determined by their age at the initiation of ERT. At week 48, the reduction in mean plasma GL-3 and Lyso-GL-3 levels in the younger group was more pronounced than in the older group (GL-3: -47.1 ± 23.6% vs. -22.0 ± 26.0%; Lyso-GL-3: -72.4 ± 17.3% vs. -48.2 ± 19.8%). Kidney function improved in the younger group (change in mean eGFR 5.2 ± 7.9 mL/min/1.73m^2^), while it declined in the older group (change in mean eGFR -5.2 ± 14.1 mL/min/1.73m^2^). The eGFR slope between the two groups (age < 30 years and age ≥ 30 years) showed a marginal difference with a P-value of 0.05, indicating that younger age may be associated with better preservation of kidney function (Supplemental Table S2 [see Additional file 1]). Furthermore, the proportion of patients experiencing symptom improvement at week 48 was higher in the younger group than in the older group (81.8% vs. 36.4%). Overall, the improvement in each estimated parameter after 48 weeks of treatment was generally greater in patients aged < 30 years than in those aged ≥ 30 years (Table 4).

**Table 4:** Subgroup analysis of age

Parameter (unit)	Age < 30 years (n = 11)	Age ≥ 30 years (n = 11)
Plasma GL-3 (ug/mL)		
Baseline	7.46 ± 2.71	6.36 ± 2.93
Week 48	3.48 ± 1.28	4.54 ± 1.71
Plasma Lyso-GL-3 (ng/mL)		
Baseline	80.35 ± 37.67	52.19 ± 36.56
Week 48	18.88 ± 12.76	22.59 ± 17.11
eGFR (mL/min/1.73m^2^)		
Baseline	140.6 ± 28.2	90.5 ± 27.4
Week 48	146.7 ± 27.3	90.0 ± 36.9
eGFR change (mL/min/1.73m^2^)	5.2 ± 7.9	-5.2 ± 14.1
Symptom improvement at Week 48, n (%)	9 (81.8)	4 (36.4)

*GL-3*, globotriaosylceramide; *Lyso-GL-3*, globotriaosylsphingosine; *eGFR*, estimated glomerular filtration rate; *n*, number

The present study overlapped with the COVID-19 pandemic; 63.6% (14/22) of the participants (11 males and 3 females) were infected with COVID-19. Among the 14 infected patients, 13 were classic patients (11 males and 2 females) and 1 was a late-onset patient (female). All infected patients reported mild to moderate infections (10 mild and 4 moderate) and subsequently recovered. No deaths or intensive care unit hospitalizations occurred due to COVID-19 infection. In the post-hoc analysis, patients were categorized into the infected (n = 14) and uninfected (n = 8) groups to explore the impact of COVID-19 infection on the prognosis of Fabry disease under ERT. Treatment compliance was comparable between the infected (74.3 ± 16.8%) and uninfected (76.0 ± 24.0%) groups. After 48 weeks of ERT, the reduction in mean plasma GL-3 and Lyso-GL-3 levels in the infected group appeared less pronounced than in the uninfected group (GL-3: -30.9 ± 28.9% vs. -41.4 ± 24.8%; Lyso-GL-3: -55.3 ± 22.7% vs. -69.7 ± 18.3%). The eGFR levels in the infected group slightly decreased after 48 weeks of treatment with a change in mean eGFR of -0.8 ± 13.6 mL/min/1.73m^2^ while the uninfected group showed improvement with a change in mean eGFR of 1.5 ± 10.6 mL/min/1.73m^2^. The comparison between eGFR slopes of the two groups (infected vs. uninfected) showed a P-value of 0.39, indicating no statistically significant difference; however, the uninfected group illustrated a more favorable trend in eGFR changes (Supplemental Table S2 [see Additional file 1]). The proportion of patients with symptom improvement at week 48 was slightly lower in the infected group than in the uninfected group (57.1% vs. 62.5%; Table 5).

**Table 5:** Subgroup analysis of COVID-19 infection

Parameter (unit)	Infected group (n = 14)	Uninfected group (n = 8)
Plasma GL-3 (ug/mL)		
Baseline	6.97 ± 2.97	6.81 ± 2.70
Week 48	4.17 ± 1.60	3.71 ± 1.58
Plasma Lyso-GL-3 (ng/mL)		
Baseline	70.74 ± 42.11	58.45 ± 34.00
Week 48	24.52 ± 15.69	13.69 ± 10.63
eGFR (mL/min/1.73m^2^)		
Baseline	114.4 ± 37.2	117.7 ± 40.1
Week 48	112.2 ± 46.2	129.8 ± 36.6
eGFR change (mL/min/1.73m^2^)	-0.8 ± 13.6	1.5 ± 10.6
Symptom improvement, n (%)	8 (57.1)	5 (62.5)

*GL-3*, globotriaosylceramide; *Lyso-GL-3*, globotriaosylsphingosine; *eGFR*, estimated glomerular filtration rate; *n*, number

## Discussion

This 54-week, open label, single arm, PMS study of agalsidase beta is the first phase 4 study to evaluate the safety and efficacy of ERT in Chinese patients with Fabry disease, providing significant insights into treatment outcomes. Despite challenges posed by the COVID-19 pandemic, the study demonstrated that ERT with agalsidase beta was well-tolerated and efficacious in reducing plasma biomarkers, improving clinical symptoms, and stabilizing kidney function in the study population. These findings underscore the importance of ERT as a key therapeutic approach for managing Fabry disease in China.

The safety endpoint was the primary endpoint of this study, and all participants (100%) experienced at least one TEAE, aligning with findings from previous studies [17, 18]. However, the COVID-19 pandemic influenced the nature of reported AEs, with COVID-19 infections accounting for 63.6% (14/22) of TEAEs, a previously undocumented phenomenon [17, 18]. SAEs were reported in 2 patients but were unrelated to the study drug. Treatment-related AEs and IARs were both reported in 36.4% (8/22) of patients. Importantly, no AEs led to permanent treatment discontinuation or death, reaffirming the safety of agalsidase beta in this population.

The incidence of IARs in this trial was 36.4% (8/22), with all categorized as mild to moderate in severity. Similar to another study [19], most first IARs occurred within initial 13 infusions, and their incidence declined over time. Notably, patients without a history of IARs did not receive pretreatment with antihistamines or analgesics, adhering to Chinese prescribing guidelines, unlike routine pretreatment in previous studies [17, 20]. Despite these differences, the IARs incidence here (36.4%) was lower than the reported 55–67% in other trials. [17, 20, 21] Several factors may explain this variance. Firstly, IARs were more frequently in male (7/18) than females (1/4), and the male proportion (81.8%) was slightly lower than in previous phase 3 (96.6%) [20] and phase 4 (88.0%) studies. [17] Secondly, the inclusion of pediatric patients (18.2%) likely influenced the results, as younger age groups had lower IAR incidences (38% in 8–16-year-olds) [22] compared to adults (55–59%) [17, 20]. Thirdly, ethnic differences might contribute, with this being the first phase 4 study to focus on Chinese patients, who may experience fewer IARs compared to European and American populations [17, 20, 23]. Finally, ADAs commonly associated with IARs in earlier studies, [24] were not assessed here, limiting conclusions. Interestingly, Mignani et al. further assessed the impact of shortening the infusion time of agalsidase beta on safety in 25 patients who had not previously received ERT or chaperone treatment, as well as 6 patients who had previously received agalsidase alfa [25]. The findings indicated that shortening the infusion time was well tolerated, with only 1 IAR in 31 patients. It is important to note that the initial infusion rate was set low, with a gradual increase over time, which may have contributed to the reduced incidence of IARs observed in this study. However, our study did not evaluate infusion time on safety, these findings highlight the need for future research to explore this aspect.

Fabry disease involves the accumulation of GL-3 and Lyso-GL-3 in cells. Evidence from the literature suggests that, in addition to diagnostic use, plasma levels of these biomarkers are widely used to monitor the response to ERT [26]. Studies have shown agalsidase beta effectively reduces plasma GL-3 and Lyso-GL-3, [11, 20] especially in male patients with classic Fabry disease with high baseline levels. In the trial, despite COVID-19 impacts, remarkable reductions were observed: plasma GL-3 decreased by -34.6 to -41.9%, and Lyso-GL-3 by -58.8 to -64.9% from baseline to week 48. Reduction was more pronounced in classic male patients, with GL-3 decreasing by 46.0% and Lyso-GL-3 by 67.8%. Subgroup analysis based on age further revealed that younger patients (< 30 years) showed greater biomarker reductions compared to older patients (≥ 30 years), emphasizing that patients initiating treatment at a younger age maximizes the therapeutic benefits, underscoring the importance of early intervention.

Symptom improvement was also evident after 48 weeks of ERT, with 59.1% (13/22) of patients reporting improved outcomes in Fabry disease symptoms. None experienced deterioration. Similarly, Borgwardt et al*.* reported improvements in headache, acroparaesthesias and gastrointestinal pain, energy levels and physical activity in children receiving agalsidase beta for 1 ~ 9 years. [27] In the subgroup analysis of age, younger patients in this study showed higher improvement rates, aligning with prior findings where patients aged 5 ~ 30 years at initiation of treatment experienced reduced symptoms after ERT, especially male [28]. These trends are suggestive of an improvement after treatment in young patients but warrant larger longitudinal studies.

The kidneys are key organs affected in Fabry disease. Research has demonstrated that ERT slows eGFR decline. A phase 3 extension study of agalsidase beta found that eGFR remained stable after 54 months. [18] Similarly, our 48-week data demonstrated stable eGFR, with an overall annual slope of 0.43 mL/min/1.73 m^2^/year (95% CI: -5.95 to 6.82) and a slightly negative slope of -0.17 mL/min/1.73 m^2^/year (95% CI: -7.71 to 7.37) in male patients. These annual slopes were slightly higher than previous reports, such as the overall population eGFR slope of -1.47 mL/min/1.73 m^2^/year (Batista et al.) [29], and the male eGFR slope of -3.4 mL/min/1.73 m^2^/year (Rombach et al.) [30], likely due to mild baseline renal involvement (18 patients with CKD G1 stage, 2 with CKD G2 stage, and 2 with CKD G3 stage), and shorter follow-up duration of 48 weeks compared to the median follow-up periods exceeding five years reported in many previous studies, and the small sample size, which may limit the statistical robustness of the results compared to larger studies. Similarly, in a real-world cohort of 125 Fabry patients with migalastat-amenable *GLA* variants receiving ≥ 3 years of migalastat treatment, the mean annualized eGFR rate of change was -0.9 (95% CI: -10.8 to 9.9) mL/min/1.73 m^2^/year (Hughes et al.) [31], further supporting the concept of disease-modifying therapies stabilizing renal function in Fabry disease. Furthermore, subgroup analysis revealed kidney function improvement in patients aged < 30 years (mean eGFR change: 5.2 ± 7.9 mL/min/1.73m^2^) but a decline in patients aged ≥ 30 years (-5.2 ± 14.1 mL/min/1.73m^2^). The difference in eGFR slope between the two age groups approached statistical significance (*P* = 0.05, Supplemental Table S2 [see Additional file 1]), suggesting that earlier intervention may be beneficial in attenuating kidney function decline. Future real-world studies with larger sample sizes are needed to validate this finding. Despite general stabilization, one patient in the present study progressed to ESRD, with eGFR from 44 mL/min/1.73m^2^ (CKD G3b stage) at baseline to 6 mL/min/1.73m^2^ (ESRD) at week 48. Notably, even in this patient with advanced renal impairment, ERT confers extrarenal benefits by reducing biomarker levels.

The implications of this study could be interpreted in light of the potential impact of the COVID-19 pandemic. This study occurred entirely during the COVID-19 pandemic, impacting patients compliance and outcomes. COVID-19 infections were the most commonly reported TEAEs (63.6%, 14/22) and infected patients showed less favorable outcomes in plasma GL-3 and Lyso-GL-3 reduction, eGFR stabilization, and symtoms compared with uninfected patients. This finding suggests that COVID-19 may be associated with a more complex renal prognosis or potentially faster disease progression despite ERT, warranting further investigation. Overall compliance (≥ 80%) was 50% (11/22), lower than 84.7% (61/72) in a prior study with hospital-based infusions [32]. Despite these challenges, agalsidase beta efficacy remained consistent with international trials, showing biomarker reductions, symptom improvement, and kidney function stability.

This study has several limitations. First, the sample size of patients included in the research is relatively small, which may impact the generalizability and robustness of the findings. Second, this study’s focus on a predominantly younger demographic and its relatively short duration (under one year) may limit the generalizability of findings to broader age groups or longer-term treatment outcomes. Third, this study primarily focuses on assessing the safety of agalsidase beta in the Chinese population, while data on efficacy remain insufficient. Specifically, future studies should consider expanding the scope of efficacy assessments to include the impact on heart and central nervous system. Additionally, the ADAs were not evaluated in this study. Future real-world studies with larger sample sizes are needed. Employing various assessment such as Mainz severity score index (MSSI) scores, to systematically evaluate patient symptoms will contribute to a more comprehensive understanding of the clinical effects of ERT. These improvements will provide a more robust basis for the application of ERT across different populations.

## Conclusions

This study marks the first phase 4 evaluation of ERT for Fabry disease in China, demonstrating that agalsidase beta is safe and well-tolerated, with a lower incidence of IARs in Chinese patients. The efficacy of agalsidase beta aligns with global studies, showing notable reductions in plasma GL-3 and Lyso-GL-3 levels, improvement in symptoms, and stabilization of kidney function. Besides, the study suggests that COVID-19 infection may potentially impact the renal prognosis in patients with Fabry disease.

## Supplementary Information

Additional file1

## Data Availability

All data generated or analysed during this study are included in this published article/Supplementary materials, further inquiries can be directed to the corresponding author on reasonable request.

## Abbreviations

α-Gal A: Alpha-galactosidase A
ADA: Anti-drug antibody
AE: Adverse event
AESI: Adverse event of special interest
CKD: Chronic Kidney Disease
eGFR: Estimated glomerular filtration rate
ECG: Electrocardiogram
ECHO: Echocardiography
EOS: End-of-study
EOT: End-of-treatment
EPI: Epidemiology Collaboration
ERT: Enzyme replacement therapy
ESRD: End stage renal disease
FDA: United States Food and Drug Administration
GCP: Good Clinical Practice
GL-3: Globotriaosylceramide
*GLA*: Galactosidase gene
IAR: Infusion-associated reaction
ICH: International Council for Harmonisation
IEC: Independent Ethics Committee
IRB: Institutional Review Board
Lyso-GL-3: Globotriaosylsphingosine
MSSI: Mainz severity score index
PMS: Post-marketing surveillance
Q2W: Every 2 weeks
SAE: Serious adverse event
SD: Standard deviation
TEAE: Treatment-emergent adverse event

## References

[1] Oder D, Müntze J, Nordbeck P. Contemporary therapeutics and new drug developments for treatment of Fabry disease: a narrative review. Cardiovasc Diagn Ther. 2021;11(2):683–95. 33968645 10.21037/cdt-20-743 PMC8102271

[2] Bertoldi G, Caputo I, Driussi G, Stefanelli LF, Di Vico V, Carraro G, et al. Biochemical mechanisms beyond glycosphingolipid accumulation in Fabry Disease: might they provide additional therapeutic treatments? J Clin Med. 2023;12(5):2063. 36902850 10.3390/jcm12052063 PMC10004377

[3] Germain DP, Altarescu G, Barriales-Villa R, Mignani R, Pawlaczyk K, Pieruzzi F, et al. An expert consensus on practical clinical recommendations and guidance for patients with classic Fabry disease. Mol Genet Metab. 2022;137(1–2):49–61. 35926321 10.1016/j.ymgme.2022.07.010

[4] Guo W, Xie Y, Ji P, Li S, Cai G, Chen X. The evolution of the initial manifestations and renal involvement of Chinese patients with classical and late-onset Fabry disease at different sexes and ages. BMC Nephrol. 2023;24(1):90. 37020293 10.1186/s12882-023-03138-w PMC10074707

[5] Arends M, Wanner C, Hughes D, Mehta A, Oder D, Watkinson OT, et al. Characterization of classical and nonclassical Fabry disease: a multicenter study. J Am Soc Nephrol. 2017;28(5):1631–41. 27979989 10.1681/ASN.2016090964 PMC5407735

[6] Ortiz A, Germain DP, Desnick RJ, Politei J, Mauer M, Burlina A, et al. Fabry disease revisited: management and treatment recommendations for adult patients. Mol Genet Metab. 2018;123(4):416–27. 29530533 10.1016/j.ymgme.2018.02.014

[7] Chinese Fabry Disease Expert Panel. Expert consensus for diagnosis and treatment of Fabry disease in China (2021). Chin J Intern Med. 2021;60(4):321–30. 10.3760/cma.j.cn112138-20201218-01028 33765701

[8] Nowicki M, Bazan-Socha S, Błażejewska-Hyzorek B, Gellert R, Imiela J, Kaźmierczak J, et al. Enzyme replacement therapy in Fabry disease in Poland: a position statement. Pol Arch Intern Med. 2020;130(1):91–7. 31868861 10.20452/pamw.15117

[9] Hopkin RJ, Jefferies JL, Laney DA, Lawson VH, Mauer M, Taylor MR, et al. The management and treatment of children with Fabry disease: a United States-based perspective. Mol Genet Metab. 2016;117(2):104–13. 26546059 10.1016/j.ymgme.2015.10.007

[10] Wanner C, Arad M, Baron R, Burlina A, Elliott PM, Feldt-Rasmussen U, et al. European expert consensus statement on therapeutic goals in Fabry disease. Mol Genet Metab. 2018;124(3):189–203. 30017653 10.1016/j.ymgme.2018.06.004

[11] van Breemen MJ, Rombach SM, Dekker N, Poorthuis BJ, Linthorst GE, Zwinderman AH, et al. Reduction of elevated plasma globotriaosylsphingosine in patients with classic Fabry disease following enzyme replacement therapy. Biochim Biophys Acta. 2011;1812(1):70–6. 20851180 10.1016/j.bbadis.2010.09.007

[12] El Dib R, Gomaa H, Ortiz A, Politei J, Kapoor A, Barreto F. Enzyme replacement therapy for Anderson-Fabry disease: a complementary overview of a Cochrane publication through a linear regression and a pooled analysis of proportions from cohort studies. PLoS ONE. 2017;12(3): e0173358. 28296917 10.1371/journal.pone.0173358 PMC5351840

[13] Chen T, Chen X, Zhang S, Zhu J, Tang B, Wang A, et al. The genome sequence archive family: toward explosive data growth and diverse data types. Genom Proteomics Bioinf. 2021;19(4):578–83. 10.1016/j.gpb.2021.08.001 PMC9039563 34400360

[14] CNCB-NGDC Members and Partners. Database Resources of the National Genomics Data Center, China National Center for Bioinformation in 2022. Nucleic Acids Res. 2022;50(D1):D27–38. 34718731 10.1093/nar/gkab951 PMC8728233

[15] Inker LA, Schmid CH, Tighiouart H, Eckfeldt JH, Feldman HI, Greene T, et al. Estimating glomerular filtration rate from serum creatinine and cystatin c. N Engl J Med. 2012;367(1):20–9. 22762315 10.1056/NEJMoa1114248 PMC4398023

[16] Schwartz GJ, Muñoz A, Schneider MF, Mak RH, Kaskel F, Warady BA, et al. New equations to estimate GFR in children with CKD. J Am Soc Nephrol. 2009;20(3):629–37. 19158356 10.1681/ASN.2008030287 PMC2653687

[17] Banikazemi M, Bultas J, Waldek S, Wilcox WR, Whitley CB, McDonald M, et al. Agalsidase-beta therapy for advanced Fabry disease: a randomized trial. Ann Intern Med. 2007;146(2):77–86. 17179052 10.7326/0003-4819-146-2-200701160-00148

[18] Germain DP, Waldek S, Banikazemi M, Bushinsky DA, Charrow J, Desnick RJ, et al. Sustained, long-term renal stabilization after 54 months of agalsidase beta therapy in patients with Fabry disease. J Am Soc Nephrol. 2007;18(5):1547–57. 17409312 10.1681/ASN.2006080816

[19] Smid BE, Hoogendijk SL, Wijburg FA, Hollak CE, Linthorst GE. A revised home treatment algorithm for Fabry disease: influence of antibody formation. Mol Genet Metab. 2013;108(2):132–7. 23332169 10.1016/j.ymgme.2012.12.005

[20] Eng CM, Guffon N, Wilcox WR, Germain DP, Lee P, Waldek S, et al. Safety and efficacy of recombinant human alpha-galactosidase A replacement therapy in Fabry’s disease. N Engl J Med. 2001;345(1):9–16. 11439963 10.1056/NEJM200107053450102

[21] Azevedo O, Gago MF, Miltenberger-Miltenyi G, Sousa N, Cunha D. Fabry disease therapy: state-of-the-art and current challenges. Int J Mol Sci. 2020;22(1): 206. 33379210 10.3390/ijms22010206 PMC7794923

[22] Wraith JE, Tylki-Szymanska A, Guffon N, Lien YH, Tsimaratos M, Vellodi A, et al. Safety and efficacy of enzyme replacement therapy with agalsidase beta: an international, open-label study in pediatric patients with Fabry disease. J Pediatr. 2008;152(4):563–70. 18346516 10.1016/j.jpeds.2007.09.007

[23] Arends M, Biegstraaten M, Wanner C, Sirrs S, Mehta A, Elliott PM, et al. Agalsidase alfa versus agalsidase beta for the treatment of Fabry disease: an international cohort study. J Med Genet. 2018;55(5):351–8. 29437868 10.1136/jmedgenet-2017-104863 PMC5931248

[24] Wilcox WR, Linthorst GE, Germain DP, Feldt-Rasmussen U, Waldek S, Richards SM, et al. Anti-α-galactosidase A antibody response to agalsidase beta treatment: data from the Fabry Registry. Mol Genet Metab. 2012;105(3):443–9. 22227322 10.1016/j.ymgme.2011.12.006

[25] Mignani R, Americo C, Aucella F, Battaglia Y, Cianci V, Sapuppo A, et al. Reducing agalsidase beta infusion time in Fabry patients: low incidence of antibody formation and infusion-associated reactions in an Italian multicenter study. Orphanet J Rare Dis. 2024;19(1):38. 38308295 10.1186/s13023-024-03049-5 PMC10835838

[26] Burlina A, Brand E, Hughes D, Kantola I, Krӓmer J, Nowak A, et al. An expert consensus on the recommendations for the use of biomarkers in Fabry disease. Mol Genet Metab. 2023;139(2): 107585. 37207471 10.1016/j.ymgme.2023.107585

[27] Borgwardt L, Feldt-Rasmussen U, Rasmussen AK, Ballegaard M, Meldgaard Lund A. Fabry disease in children: agalsidase-beta enzyme replacement therapy. Clin Genet. 2013;83(5):432–8. 22880956 10.1111/j.1399-0004.2012.01947.x

[28] Hopkin RJ, Cabrera GH, Jefferies JL, Yang M, Ponce E, Brand E, et al. Clinical outcomes among young patients with Fabry disease who initiated agalsidase beta treatment before 30 years of age: an analysis from the Fabry Registry. Mol Genet Metab. 2023;138(2): 106967. 36709533 10.1016/j.ymgme.2022.106967

[29] Batista JL, Hariri A, Maski M, Richards S, Gudivada B, Raynor LA, et al. Reduction in kidney function decline and risk of severe clinical events in agalsidase beta-treated Fabry disease patients: a matched analysis from the Fabry Registry. Clin Kidney J. 2024;17(8):sfae194. 10.1093/ckj/sfae194. 39139182 10.1093/ckj/sfae194 PMC11320591

[30] Rombach SM, Smid BE, Bouwman MG, Linthorst GE, Dijkgraaf MG, Hollak CE. Long term enzyme replacement therapy for Fabry disease: effectiveness on kidney, heart and brain. Orphanet J Rare Dis. 2013;8:47. 23531228 10.1186/1750-1172-8-47 PMC3626869

[31] Hughes DA, Sunder-Plassmann G, Jovanovic A, Brand E, West ML, Bichet DG, et al. Renal and multisystem effectiveness of 3.9 years of migalastat in a global real-world cohort: results from the followme Fabry pathfinders registry. J Inherit Metab Dis. 2025;48(1): e12771. 39031114 10.1002/jimd.12771 PMC11730455

[32] Concolino D, Amico L, Cappellini MD, Cassinerio E, Conti M, Donati MA, et al. Home infusion program with enzyme replacement therapy for Fabry disease: the experience of a large Italian collaborative group. Mol Genet Metab Rep. 2017;12:85–91. 28702361 10.1016/j.ymgmr.2017.06.005 PMC5484973

