# Supplementary material for: A phase 4, open-label, multicenter study of the safety and efficacy of agalsidase beta in Chinese patients with Fabry disease
Source: Orphanet J Rare Dis. 2025 Aug 4;20:401. doi: 10.1186/s13023-025-03950-7 (PMC12323282; doi:10.1186/s13023-025-03950-7)
Supplement: Supplementary file 1 — Additional file1 [file 13023_2025_3950_MOESM1_ESM.docx]

**Study sites:**

| No. | Study sites |
| --- | --- |
| 1 | Ruijin Hospital, The Medical School of Shanghai Jiao Tong University |
| 2 | Peking Union Medical College Hospital |
| 3 | Peking University First Hospital |
| 4 | Children's Hospital of Fudan University |
| 5 | Tongji Hospital, Tongji Medical College, Huazhong University of Science and Technology |
| 6 | First Hospital of Shanxi Medical University |

**Inclusion Criteria**

Participants are eligible to be included in the study only if all of the following criteria apply:

**Age**

I 01. Participant must be 8 years of age or older, at the time of signing the informed consent.

**Type of participant and disease characteristics.**

I 02. Participants naive to agalsidase beta and agalsidase alpha.

I 03. Chinese participants diagnosed with Fabry disease and with documented plasma or leukocyte αGAL activity deficient below laboratory’s reference range, and/or documented diagnosis by genotyping.

I 04. Participants must have one or more symptoms and signs consistent with manifestations of Fabry disease (not limited to neuropathic pain, chronic kidney disease, hypertrophic cardiomyopathy, cardiac rhythm disturbances, cerebrovascular involvement, cornea verticillata, angiokeratoma, gastrointestinal symptoms, hypo- or anhydrosis).

**Sex, contraceptive/barrier method and pregnancy testing requirements**

I 05. Male and female

A female participant is eligible to participate if she is not pregnant or breastfeeding, and at least one of the following conditions applies:

- Is not a woman of childbearing potential (WOCBP)

OR

- Is a WOCBP and agrees to use an acceptable contraceptive method as described in Appendix 4 (Section 10.4) during the intervention period and at a minimum until 14 days after the last dose of study intervention.

- A WOCBP must have a negative highly sensitive pregnancy test (urine or serum as required by local regulations) within the screening period before the first dose of study intervention.

- If a urine test cannot be confirmed as negative (eg, an ambiguous result), a serum pregnancy test is required. In such cases, the participant must be excluded from participation if the serum pregnancy result is positive.

- Requirements for pregnancy testing during and after study intervention are located in the SoA (Section 1.3).

**Informed Consent**

I 06. Participants and/or participant’s legal representative capable of giving signed informed consent as described in Appendix 1 (Section 10.1) which includes compliance with the requirements and restrictions listed in the informed consent form (ICF) and in this protocol. For potential participants aged 8 to <18 years, a parent or legal representative is required to sign the ICF, and the potential participant is also required to sign an informed assent form.

I 07. The participant has to be able to comply with the clinical protocol, which required extensive clinical evaluations.

**Exclusion Criteria**

Participants are excluded from the study if any of the following criteria apply:

**Medical conditions**

E 01. The participant has undergone kidney transplantation.

E 02. The participant has a clinically significant organic disease (with the exception of symptoms relating to Fabry disease) in the opinion of the Investigator, would preclude participation in the trial.

**Prior/concomitant therapy**

E 03. Received substrate reduction therapy within 30 days of anticipated administration of IMPs or 5 half-lives of the previous treatment, whichever is longer.

**Prior/concurrent clinical study experience**

E 04. Received an investigational drug, or device, other than Fabrazyme, within 30 days of anticipated IMPs administration or 5 half-lives of the previous investigational drug, whichever is longer.

E 05. Received an investigational gene therapy.

**Diagnostic assessments**

E 06. The patient has current evidence of kidney failure or renal insufficiency, as defined by eGFR <30 mL/min/1.73 m^2^.

**Other exclusions**

E 07. Individuals who have life threatening hypersensitivity (anaphylactic reaction) to the active substance or any of the excipients included.

E 08. Individuals accommodated in an institution because of regulatory or legal order; prisoners or participants who are legally institutionalized.

E 09. Participant not suitable for participation, whatever the reason, as judged by the Investigator, including medical or clinical conditions, or participants potentially at risk of noncompliance to study procedures.

E 10. Participants are employees of the clinical study site or other individuals directly involved in the conduct of the study, or immediate family members of such individuals (in conjunction with Section 1.61 of the ICH-GCP Ordinance E6).

E 11. Any specific situation during study implementation/course that may rise ethics considerations.

E 12. Sensitivity to any of the study interventions, or components thereof, or drug or other allergy that, in the opinion of the Investigator, contraindicates participation in the study.

**An LC-MS/MS Method for the Determination of Plasma GL-3**

Sample Preparation:

After vortexing the samples, transfer 20.0 µL of plasma into a 2.2 mL 96-well plate (e.g., Apricot Designs). For all samples except double blanks, add 40.0 µL of internal standard (IS) working solution; for double blanks, add 40.0 µL of methanol (MeOH). Centrifuge the plate at 10,000 g for 10 minutes at 4 °C. Add 340 µL of MeOH to each well, seal the plate with aluminum sealing film, and vortex thoroughly. Centrifuge again under the same conditions and transfer 200 µL of the supernatant to a new 96-well plate. Ensure proper sealing with a mat to avoid evaporation or contamination during subsequent steps.

Chromatographic Separation:

The samples are loaded onto solid-phase extraction columns and eluted with 1 mL of 9:1 acetone:MeOH. The eluates are dried under nitrogen and reconstituted with 100 µL of MeOH before injection. Chromatographic separation is performed on a C8 analytical column (alternative options such as C18 or HILIC may be considered for improved isomer resolution if needed).

Mass Spectrometry Analysis:

The reconstituted samples are analyzed by LC-MS/MS in multiple reaction monitoring (MRM) mode. Ten major GL-3 isomers (C16:0, C18:0, C20:0, C22:1, C22:0, C22:0-OH, C24:1, C24:0, C24:0-OH, and C26:0) are quantified using a C17:0 GL-3 analog as the internal standard. Specific precursor-to-product ion transitions are monitored for each isomer to ensure accurate quantification.

Calibration and Quantification:

The total GL-3 concentration in plasma samples is determined by dividing the GL-3/IS peak area ratio by the slope of the calibration curve. A linear calibration curve is established over a range of concentrations that encompass expected patient values, with an R² > 0.99. The lower limit of quantitation (LLOQ) for the assay is 800 ng/mL; sensitivity improvements to 200 ng/mL are achievable if needed for clinical samples with low GL-3 levels.

**An LC-MS/MS Method for the Determination of Plasma Lyso-GL-3**

Sample Preparation:

Vortex the plasma samples thoroughly. Transfer 50.0 µL of plasma into a 96-well plate. For all samples except double blanks, add 25.0 µL of internal standard (IS) working solution; for double blanks, add 25.0 µL of methanol (MeOH). Mix the samples by gentle pipetting or vortexing to ensure complete incorporation of the IS or MeOH. Centrifuge the plate at 10,000 g for 10 minutes at 4 °C. Add 325 µL of MeOH to each well to precipitate proteins, seal the plate with aluminum sealing film, and vortex the plate for 1 minute at 1,200 rpm. Centrifuge the plate again under the same conditions. Carefully transfer 200 µL of the supernatant to a new 96-well plate to avoid disturbing the precipitate.

Pre-Analysis Treatment:

Add 50.0 µL of 1% formic acid (FA) in water to each well to adjust the pH and enhance ionization efficiency during LC-MS/MS analysis. Seal the plate with a mat, and vortex it for about 1 minute at 1,200 rpm to ensure proper mixing.

Chromatographic and Mass Spectrometry Analysis:

Load the prepared plate onto the autosampler of the LC-MS/MS system. Use a suitable analytical column (e.g., C18 or C8) to achieve efficient separation of Lyso-GL-3 from other components. Quantification is performed in multiple reaction monitoring (MRM) mode, with optimized precursor-to-product ion transitions for Lyso-GL-3 and the internal standard.

Calibration and Quantification:

The concentration of Lyso-GL-3 in patient samples is calculated using the ratio of Lyso-GL-3 peak area to the IS peak area, divided by the slope of a linear calibration curve. The calibration curve is constructed over a clinically relevant range with an R² > 0.99. The lower limit of quantification (LLOQ) for the assay is 0.140 ng/mL, validated to meet regulatory guidelines for sensitivity and accuracy.

**Supplemental Table S1. Number (%) of participants experiencing at least one treatment emergent AESI.**

| **AESIs** | **Agalsidase beta (N=22)** |
| --- | --- |
| IARs | 8 (36.4) |
| Feeling hot | 3 (13.6) |
| Pyrexia | 3 (13.6) |
| Nausea | 2 (9.1) |
| Pruritus | 2 (9.1) |
| Chest discomfort | 2 (9.1) |
| Vomiting | 1 (4.5) |
| Rash | 1 (4.5) |
| Myalgia | 1 (4.5) |
| Pain in extremity | 1 (4.5) |
| Chills | 1 (4.5) |

*AESI*, adverse events of special interest; *IAR*, infusion associated reaction; *N*, number.

**Supplemental Table S2. eGFR slope of each group in age subgroup (Age <30 years and Age ≥30 years) and COVID-19 infection subgroup (Infected group and Uninfected group)**

| **Parameter** | **Age <30 years (n=11)** | | **Age ≥30 years (n=11)** | | **P** |
| --- | --- | --- | --- | --- | --- |
| eGFR Slope (mL/min/1.73m^2^/year) (95%CI) | n=11 | 5.88 (-3.53, 15.29) | n=10 | -5.08 (-17.70, 7.55) | 0.05 |
| **Parameter** | **Infected group (n=14)** | | **Uninfected group (n=8)** | | **P** |
| eGFR Slope (mL/min/1.73m^2^/year) (95%CI) | n=14 | -1.03 (-9.86, 7.80) | n=7 | 4.50 (-10.44, 19.44) | 0.39 |

*Note: There was one male patient over 30 years old who was not infected with COVID-19 and was lost to follow-up during the subsequent treatment period, so that he was not involved in the calculation of eGFR slope.*

*eGFR*, estimated glomerular filtration rate; n, number.

**Supplemental Figure S1. a) Scatter plot of plasma GL-3 changes from baseline to week 48 in male patients; b) Scatter plot of plasma GL-3 changes from baseline to week 48 in female patients.**


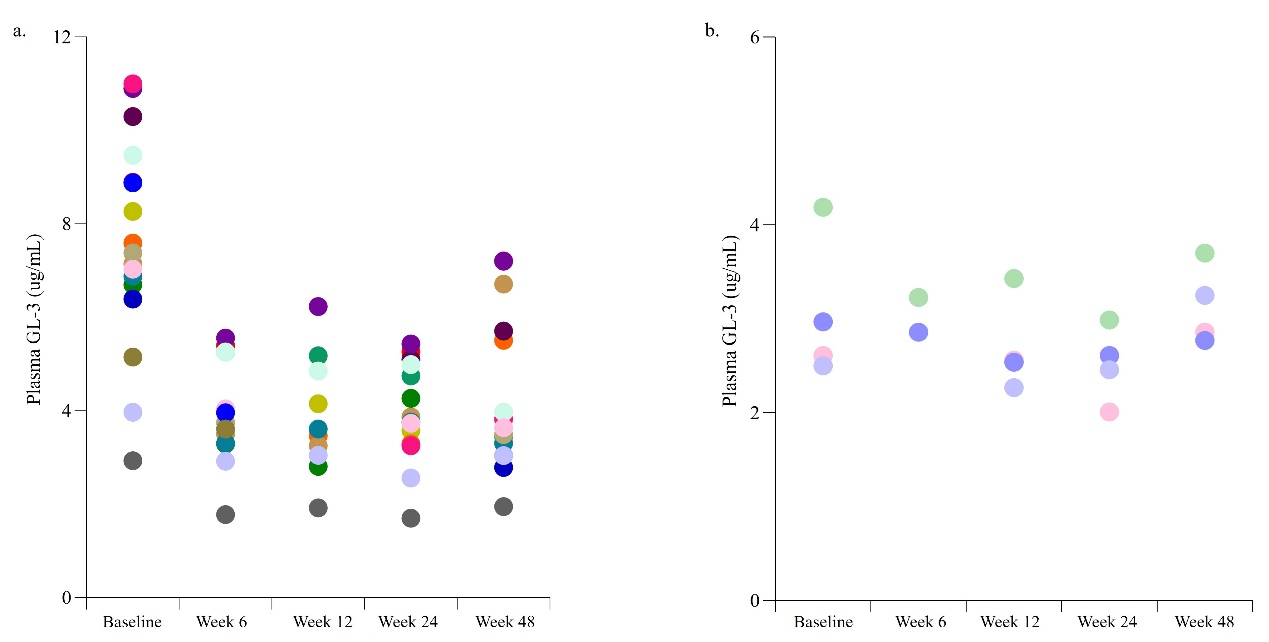


*GL-3*, globotriaosylceramide.

**Supplemental Figure S2. a) Scatter plot of plasma Lyso-GL-3 changes from baseline to week 48 in male patients; b) Scatter plot of plasma Lyso-GL-3 changes from baseline to week 48 in female patients.**


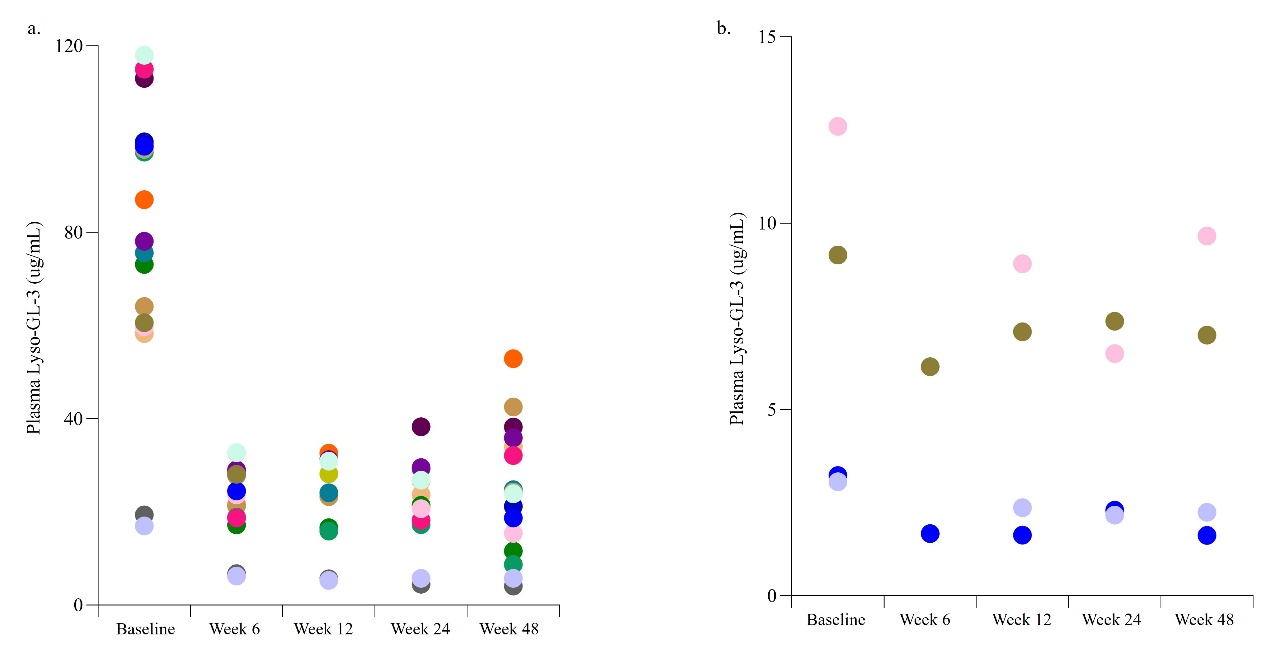


*Lyso-GL-3*, globotriaosylsphingosine.

**Supplemental Figure S3. a) Scatter plot of eGFR changes from baseline to week 48 in male patients; b) Scatter plot of eGFR changes from baseline to week 48 in female patients.**


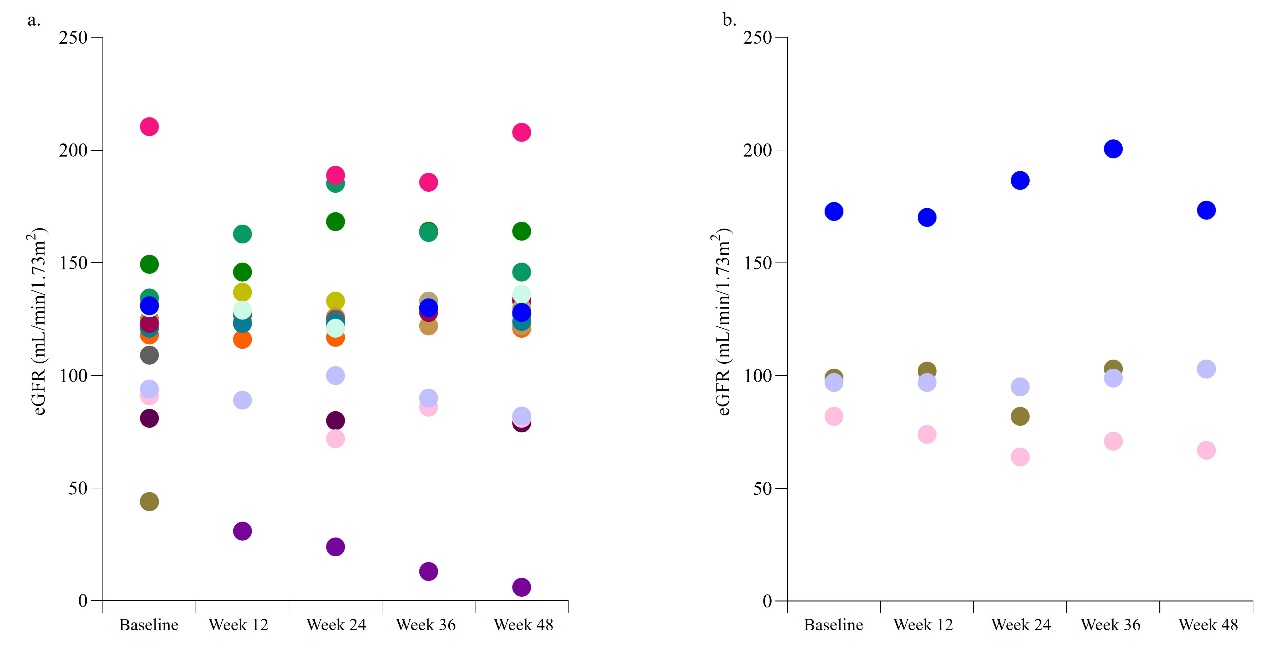


*eGFR*, estimated glomerular filtration rate.

**Supplemental Figure S4. eGFR changes after 48 weeks of treatment in groups based on baseline eGFR levels.**


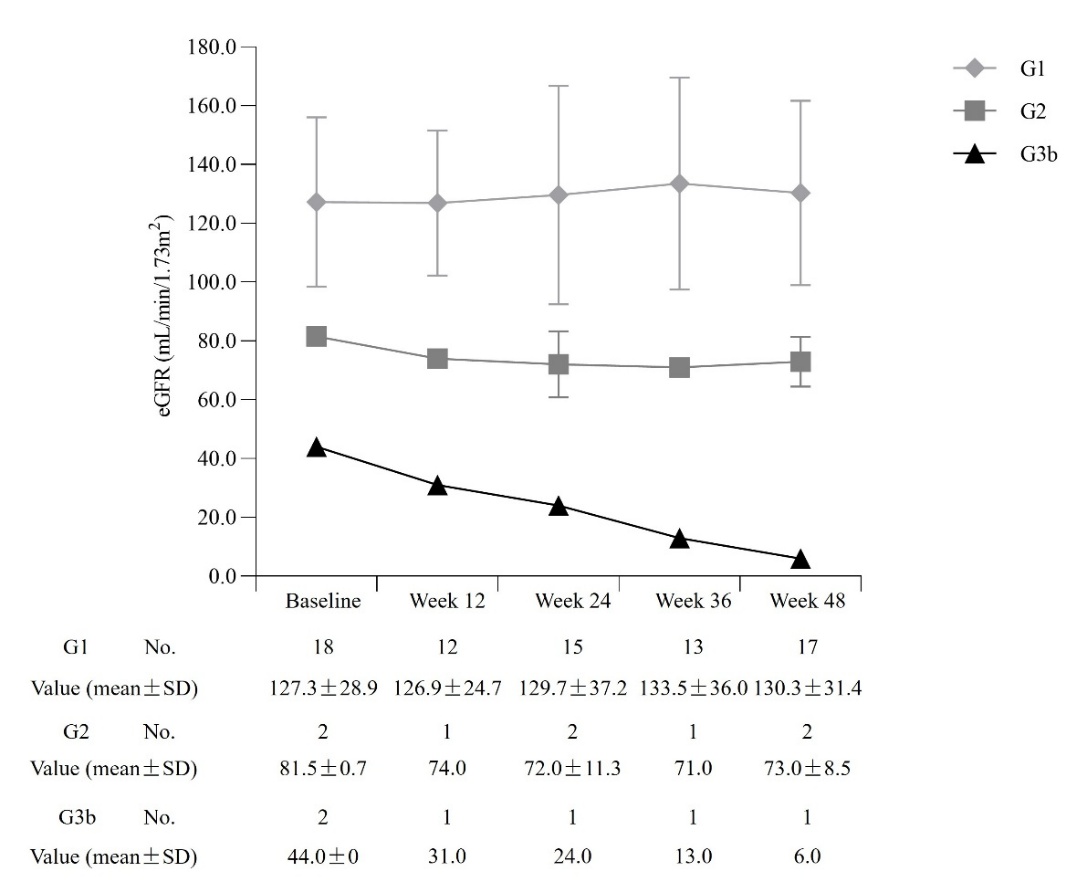


*Note: At baseline, there were two patients in each of the G2 and G3 stages of Chronic Kidney Disease (CKD). In the CKD G2 stage group, one patient was lost to follow-up at week 12 and week 36. In the CKD G3 stage group, one patient was lost to follow-up at weeks 12, 24, 36, and 48, the other ultimately progressed to end-stage renal disease (ESRD), whose eGFR progressively decreased from 44 mL/min/1.73m^2^ (CKD G3b stage) at baseline to 6 mL/min/1.73m^2^ (ESRD) at week 48. After agalsidase beta treatment, the reduction pattern of the plasma GL-3 (10.9 mg/L at baseline and 7.2 mg/L at week 48; -33.9%) and Lyso-GL-3 (78.1 ng/mL at baseline and 35.9 ng/mL at week 48; -54.0%) in this patient was similar to that observed in the overall study population. Additionally, the Fabry disease specific symptoms of this patient remained unchanged during the study.*

*eGFR*, estimated glomerular filtration rate; *No.*, number; *SD*, Standard deviation.
